# Superenhancers as master gene regulators and novel therapeutic targets in brain tumors

**DOI:** 10.1038/s12276-023-00934-0

**Published:** 2023-02-01

**Authors:** Hai-Hui Zhuang, Qiang Qu, Xin-Qi Teng, Ying-Huan Dai, Jian Qu

**Affiliations:** 1 Department of Pharmacy, the Second Xiangya Hospital, Central South University, Institute of Clinical Pharmacy, Central South University, Changsha, 410011 PR China; 2 Department of Pharmacy, Xiangya Hospital, Central South University, Changsha, 410007 PR China; 3 Institute for Rational and Safe Medication Practices, National Clinical Research Center for Geriatric Disorders, Xiangya Hospital, Central South University, Changsha, 410007 PR China; 4 Department of Pathology, the Second Xiangya Hospital, Central South University, Changsha, 410011 PR China

**Keywords:** CNS cancer, Transcriptional regulatory elements

## Abstract

Transcriptional deregulation, a cancer cell hallmark, is driven by epigenetic abnormalities in the majority of brain tumors, including adult glioblastoma and pediatric brain tumors. Epigenetic abnormalities can activate epigenetic regulatory elements to regulate the expression of oncogenes. Superenhancers (SEs), identified as novel epigenetic regulatory elements, are clusters of enhancers with cell-type specificity that can drive the aberrant transcription of oncogenes and promote tumor initiation and progression. As gene regulators, SEs are involved in tumorigenesis in a variety of tumors, including brain tumors. SEs are susceptible to inhibition by their key components, such as bromodomain protein 4 and cyclin-dependent kinase 7, providing new opportunities for antitumor therapy. In this review, we summarized the characteristics and identification, unique organizational structures, and activation mechanisms of SEs in tumors, as well as the clinical applications related to SEs in tumor therapy and prognostication. Based on a review of the literature, we discussed the relationship between SEs and different brain tumors and potential therapeutic targets, focusing on glioblastoma.

## Brain cancer: opportunities to block “super-enhanced” tumor growth

“Superenhancer” regions within the genome help coordinate the malignant growth of brain tumors, and could offer a target for the development of therapies and prognostic biomarkers. These regions contain a dense accumulation of gene regulatory sequences, which operate in collaboration to establish cellular identity. A review by Jian Qu and colleagues at Central South University, Changsha, China, shows how these regulatory sequences get hijacked in various brain cancers and can facilitate tumor cell proliferation and invasion. Many tumor cells acquire mutations that lead to the formation of superenhancers that are absent in healthy cells. However, this process also offers therapeutic opportunities, and researchers have identified numerous drug candidates that can potentially block tumorigenesis by selectively interfering with superenhancer function. Superenhancer activity could also provide a potentially useful clinical signature for assessing tumor aggressiveness.

## Introduction

Brain tumors, including glioblastoma, medulloblastoma, diffuse intrinsic pontine glioma, meningioma, ependymoma, and other rare brain tumors, account for approximately 3% of cancer cases worldwide^1^. While brain tumors are relatively rare, they still deserve research attention because of their significant mortality and morbidity at all ages^2^. Currently, treatments for brain tumors include surgery, chemotherapy, and radiotherapy. Despite substantial advances in chemotherapy for different brain tumors, chemotherapy resistance and relapse are still challenges. Delivering therapeutic agents to primary brain tumors is particularly challenging because of the unique brain–blood barrier (BBB) and brain–tumor barrier (BTB), resulting in poor response to treatments^3,4^. Moreover, brain tumor cells breach the BBB and adhere to the BTB, and this diffuse mode of invasion is the fundamental reason for brain metastases and relapse^4^. Therefore, it is necessary to study pathogenesis further and find more effective targets to improve the prognosis of patients with brain tumors.

Gene transcription is a complex and highly coordinated process. Transcriptional dysregulation mediated by epigenetic modifications in tumors has attracted significant attention. Over the last decades, epigenetic modifications such as DNA methylation and histone modification have been identified as critical drivers of several types of brain cancer^5^. Enhancers are a class of regulatory DNA sequences that function as *cis*-regulatory elements to enhance the transcription of target genes occupied by coactivators and transcription factors (TFs)^6,7^. With the expanding concept of enhancers, “superenhancers (SEs)” have been proposed, which span several kilobases and are enriched with a higher density of TFs, coactivators, and epigenetic modifications^8^. Compared to typical enhancers, SEs can drive higher gene expression and participate in many biological processes^9^. It is worth mentioning that oncogenes regulated by SEs in tumor cells are not expressed in normal cells, suggesting that SEs play a critical role in the occurrence and development of tumors^10^. As tumor-associated variants are significantly enriched in SEs, identifying SEs improves the understanding of the mechanisms of tumorigenesis and provides new insights into the diagnosis, treatment, and prognosis of tumors^11^. Overall, SE-driven transcriptional disorders are associated with the progression of human cancers, including brain tumors.

This review will summarize the characteristics and identification, unique organizational structures, and activation mechanisms of SEs in tumors, as well as the clinical applications related to SEs in tumor therapy and prognostication. Furthermore, we will discuss SE-associated genes, transcriptional regulatory mechanisms, and therapeutic targets in different brain tumors.

## Overview of superenhancers

### Characteristics and identification of SEs

In 2013, Young et al. first proposed superenhancers: large clusters of transcriptional enhancers that drive the expression of cell-identity genes^10^. SEs were initially discovered in embryonic stem cells (ESCs), with ESC-specific master transcription factors Oct4, Sox2, and Nanog binding to enhancer elements and recruiting mediators to activate the gene transcription program^12^. Subsequently, SEs were identified in other cells, including cancer cells^13,14^. As novel epigenetic regulatory elements, SEs precisely regulate oncogene transcriptional activation during tumorigenesis and may be potential therapeutic targets^15,16^.

**Fig. 1:**
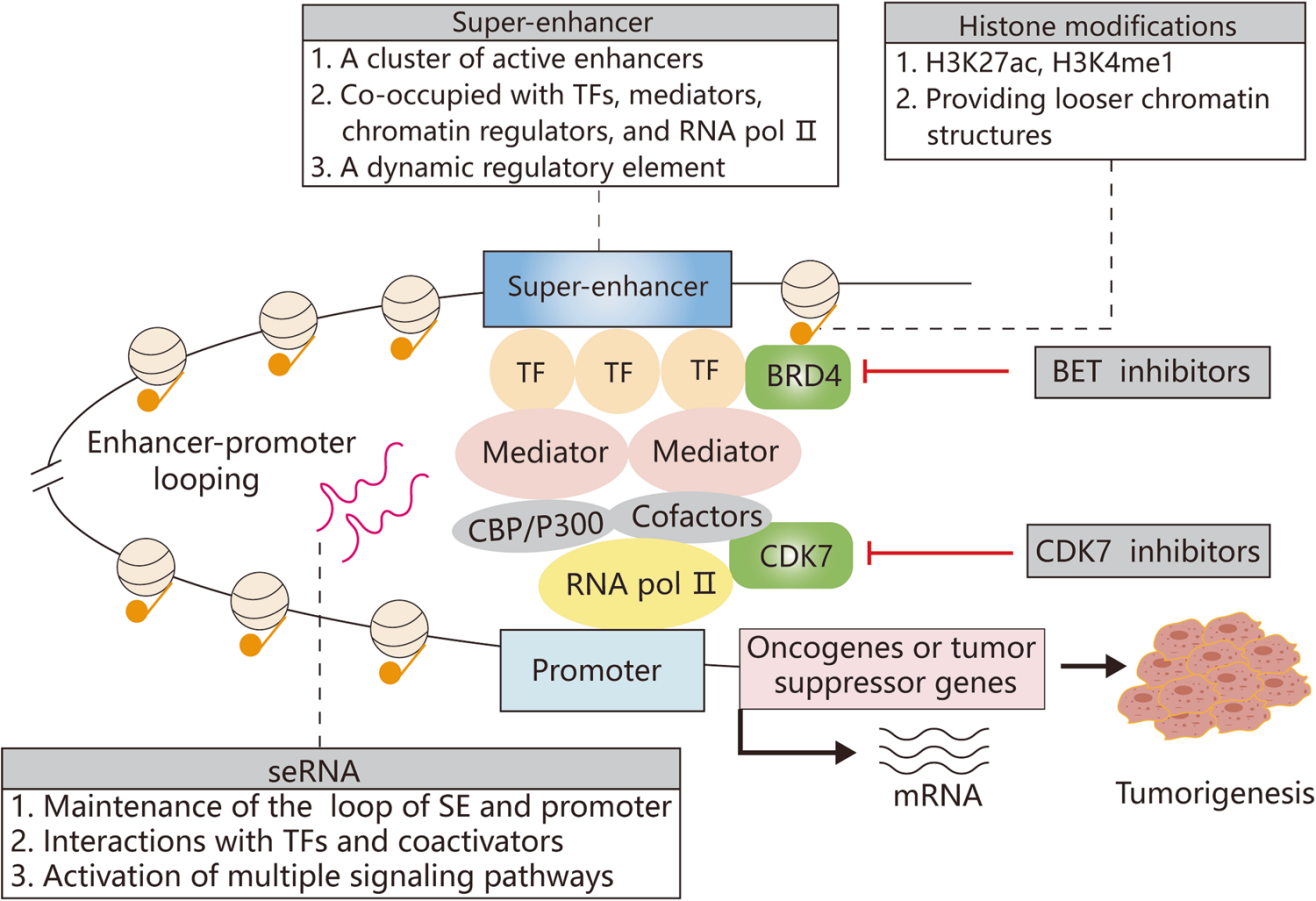
Schematic diagram of the structure and characteristics of SEs. SEs recruit TFs, mediators, RNA pol II, histone modifiers, and other chromatin regulators, activating the expression of downstream genes. The enhancer-promoter loop that is formed with the help of seRNAs contributes to the transcription of target genes. Blocking SEs with BRD4 or CDK7 inhibitors is considered a viable antitumor approach. SE superenhancer, TF transcription factors, BRD4 bromodomain protein 4, CDK7 cyclin-dependent kinase 7.

SEs are bound by many cell-type-specific TFs and recruit a series of mediators, transcriptional coactivators, and chromatin regulators (CBP/p300 and cohesin) as well as RNA polymerase II (RNA pol II), thus forming an SE-promoter loop to initiate downstream transcription^17^. In addition, histone modifications are important features of SEs. Histone H3 lysine 27 acetylation (H3K27ac) and histone H3 lysine 4 methylation (H3K4me1), which make the chromatin structure looser and provide accessible TF binding sites, are labels of active SEs^18,19^. H3K27ac and H3K4me1 are required for enhancers to activate target gene transcription, and these active chromatin markers can mediate the recruitment of epigenetic readers, including BET proteins^20,21^. In conclusion, these key components are integral parts of SE organization and the functional basis of SEs (Fig. 1).

For the identification of SEs, next-generation sequencing and high-throughput sequencing technologies, including chromatin immunoprecipitation sequencing (ChIP-seq)^22^, DNase I sequencing (DNase-seq)^23^, chromosome conformation capture sequencing (3C-seq)^24^, and assay for transposase-accessible chromatin sequencing (ATAC-seq), are powerful tools^25^. SEs can be identified based on the strength of their binding with chromatin regulators and histone modification levels. Histone modifications can be used as indicators to identify SEs based on genome-wide factors such as the ChIP-seq-identified level of H3K27ac. Moreover, as an increasing number of SEs are defined in different types of tumors, various databases have been established to explore SE functions and facilitate SE research, such as SEdb (http://www.licpathway.net/sedb) and dbSUPER (http://bioinfo.au.tsinghua.edu.cn/dbsuper/)^26–40^.

### The role of SE-derived RNAs

Enhancers regulate the transcription of target genes and are actively transcribed into enhancer RNAs (eRNAs), and this ability to be transcribed is a general feature of functionally active enhancers^41^. Correspondingly, SEs are also transcribed into seRNAs, including long noncoding RNAs (lncRNAs), microRNAs (miRNAs), and circular RNAs (circRNAs), which play an important role in gene expression and epigenetic regulation^42–44^.

A growing number of studies have revealed the critical roles of seRNAs in regulating target gene transcription. Young’s group proposed an RNA-mediated feedback control model of transcriptional condensates. In the model, low levels of RNAs mediate the formation of transcriptional condensates, while high levels of RNAs cause condensate dissolution, thus affecting the transcriptional output^45^. The seRNAs act synergistically with SEs to regulate gene expression by maintaining the SE-promoter chromatin loop^46^. For example, the seRNA HPSE interacts with hnRNPU and p300 and then facilitates chromatin looping between the SE and HPSE promoter to enhance HPSE expression, thus promoting cancer progression^47^.

Although the role of seRNAs in transcriptional regulation in both *cis* and *trans* is clear, the molecular mechanism is still controversial. Three models of seRNA mechanisms have been reported: interacting with TFs or coactivators, driving enhancer-promoter looping, and transferring to the cytoplasm to mediate various cell activities^48^. While most studies have demonstrated that seRNAs promote enhancer-promoter looping, Panigrahi *et al*. showed that seRNAs do not affect chromatin looping^49^. They concluded that mutual costimulation of enhancer and promoter transcription is not dependent on seRNAs^49^. Further studies are required to explain these conflicting observations regarding the molecular mechanism of seRNAs.

SeRNAs that are generated from genetic alterations or somatic mutations of SEs during tumorigenesis and are termed oncogenic seRNAs^46^ (Fig. 1). Oncogenic seRNAs are significantly upregulated in several types of tumors and associated with carcinogenic processes, including cell proliferation, apoptosis, autophagy, and epithelial-mesenchymal transition (EMT). In hepatocellular carcinoma (HCC), HCCL5, an SE-driven lncRNA, is significantly overexpressed and promotes cell growth, invasion, and metastasis^50^. Recently, the SE-lncRNA FASRL has been identified in HCC, and FASRL can increase fatty acid synthesis and lipid accumulation, thereby exacerbating HCC progression^51^. The novel SE-lncRNA LOC100506178 was found to promote nasopharyngeal carcinoma metastasis by interacting with the TF hnRNPK and controlling the expression of hnRNPK, accelerating the EMT process^52^. In addition, SEs can promote the transcription of pri-miRNAs and recruitment of the Drosha/Dgcr8 complex, thereby increasing the expression of cell-specific miRNAs^43^. In HCC, the YY1/p65/p300 complex increases QK1 expression through interaction with the SE-promoter loop, thus promoting HCC malignancy and increasing circRNA formation and EMT^53^. SEs usually contain discrete loci with seRNA expression peaks, and seRNA signals in cancer samples have been identified to have clinical relevance^54^. Although some studies have reported a correlation between SE-derived ncRNAs and prognostic indicators, most were meta-analyses, and thus, further clinical validation is required^55^.

### Unique organizational structures of SEs

Studies on SEs consider not only the linear structure of the genome but also the three-dimensional (3D) structure of the genome. In the eukaryotic genome, DNA‒protein complexes fold 3D chromatin loops, called topologically associating domains (TADs), which are structural units for transcriptional regulation^56^. SEs can be enriched in the chromatin loop inside TADs, and SE-containing TADs have more chromatin interactions^57^. TADs insulate promoters from enhancers and superenhancers, and the disruption of TADs alters regulatory circuits and leads to oncogene activation^58,59^. The architectural protein CTCF interacting with the cohesin complex defines the boundary for TADs and SEs, and this interaction is specifically required for the formation of chromatin loops, allowing orderly gene regulation^58,60,61^. In T-cell acute lymphoblastic leukemia, loss of CTCF induces a TAD fusion event, which leads to a direct interaction between the MYC promoter and a distal SE, thus activating MYC^62^. Especially in the absence of cohesin, SEs tend to form links with each other, resulting in extensive SE fusion^63^. Therefore, an accurate understanding of the 3D genome chromatin structure is essential for SE-mediated transcriptional regulation.

**Fig. 2:**
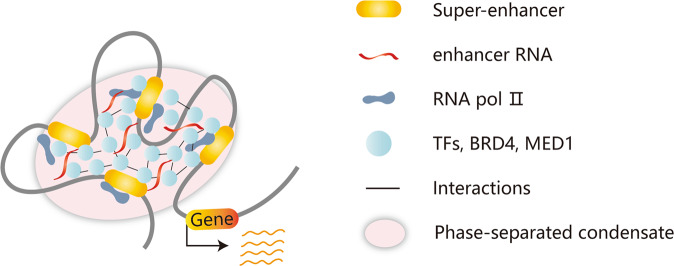
The liquid–liquid phase separation model of SEs. At the superenhancer locus, transcriptional regulators with extensive interactions, including TFs, BRD4, MED1, RNA pol II, and enhancer RNAs, are enriched to form a phase-separated condensate, which is separated from other chromatin domains and can drive transcriptional bursting and produce simultaneous activation of genes.

Liquid–liquid phase separation (LLPS), a dynamic physicochemical process, forms a membraneless organelle termed a condensate to organize biological processes within cells^64^. In 2017, the proposed phase separation model limited SE-specific gene regulation to the membraneless organelle^65^. The intrinsically disordered regions (IDRs) of TFs, the transcriptional coactivators bromodomain protein 4 (BRD4) and mediator complex subunit 1 (MED1), and RNA Pol II can form phase-separated condensates at SE regions, leading to the transcriptional bursting and simultaneous activation of SE-driven genes^66,67^. The phase-separated condensate can separate SEs from other chromatin regions and concentrate SE-associated transcriptional processes at key regions, which explains why the effect of transcription regulation of SEs is greater than the overall effect of individual typical enhancers^66^. In leukemias, NUP98–HOXA9, a NUP98 fusion oncoprotein containing IDRs, can form LLPS condensates. LLPS of NUP98–HOXA9 is critical for leukemogenesis because it not only induces CTCF-independent chromatin loops but also leads to the formation of a “superenhancer”-like binding pattern at leukemogenic genes to regulate transcriptional activity^68,69^. The pharmacological inhibition of LLPS condensates can suppress metastasis and chemoresistance in osteosarcoma, representing a novel therapeutic strategy^70^. In addition, seRNAs also contribute to the organization of the phase-separated condensate and play a role in transcriptional activation^71^. Current studies on phase separation mainly focus on the role of biomolecular condensates assembled in tumors. However, such studies have not considered the intrinsic mechanism of the dynamic phase separation process, which still needs further investigation. Therefore, the phase separation model, which can ensure the precise regulation of genes, provides a novel perspective to elucidate the formation and transcriptional regulatory mechanisms of SEs (Fig. 2).

The gene transcription process requires a variety of transcriptional activators at specific DNA regulatory elements. To coordinate transcription programs in normal and malignant cells, a small group of master TFs forms an interconnected core regulatory circuitry (CRC) by directly co-occupying their SEs and each other’s SEs, allowing close contact between SEs and their target promoters^8,72^. The activities of SEs and TFs affect each other. On the one hand, SEs coordinate with TFs to regulate the gene expression program, and the activity of SEs is affected by TF enrichment. On the other hand, the expression of TFs is often regulated by the activity of SEs, indicating positive feedback regulation between SEs and TFs^8^. The interconnected autoregulatory loop containing SEs can regulate specific cell-type transcriptional programs. To date, the CRC model and associated master TFs have been identified in multiple cancer types, including esophageal cancer^72^, acute myeloid leukemia^73^, multiple myeloma^74^, and osteosarcoma^70^. Because of the important role of CRC in malignant tumors, targeting the core TFs may suppress tumor growth.

### Functional activation of SEs during tumorigenesis

**Fig. 3:**
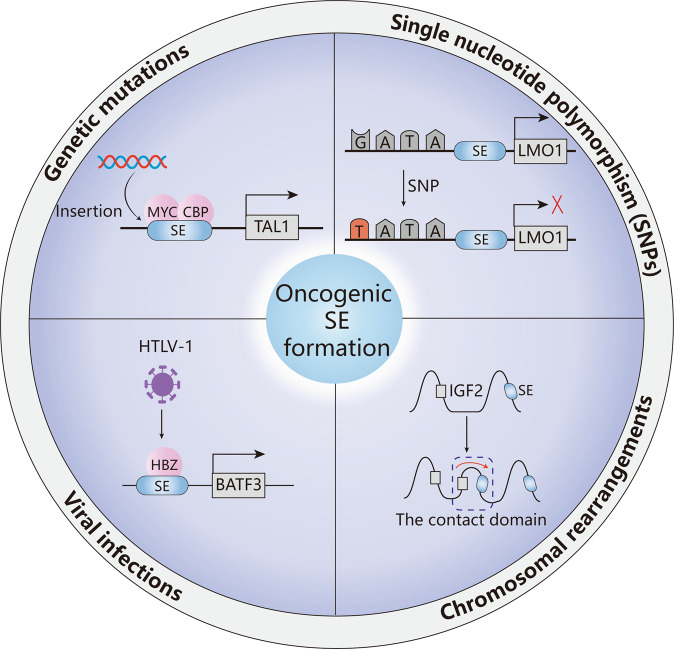
Functional activation mechanisms of oncogenic SEs. Genetic mutations, single-nucleotide polymorphisms (SNPs), chromosomal rearrangements, and viral infections lead to SE formation and oncogene activation.

Cancer cells proactively construct SEs via mechanisms such as genetic mutation, single-nucleotide polymorphism (SNP), chromosomal rearrangement, and viral infection^11^ (Fig. 3). During tumorigenesis, the functional activation of SEs leads to the dysregulation of transcriptional programs, making tumors highly dependent on gene expression regulators^75^.

Genetic mutations lead to SE activation by creating new binding sites for TFs, changing SE copy numbers, and changing the spatial structure of the genome. In T-cell acute lymphoblastic leukemia cells, there is a 12 bp insertion upstream of the *TAL1* oncogene, which generates an SE region through the binding of MYC and the recruitment of CBP and other core TFs, driving the aberrant expression of key oncogenes^76^ (Fig. 3). In addition to insertion, focal amplification of SEs is a common mechanism of SE activation. For instance, two focal amplifications of SEs located on the 3’ side of *MYC* in endometrial carcinoma and lung cancer are close to the *MYC* promoter region and are associated with aberrant expression of *MYC*^77^.

Some specific tumor-related SNPs in regulatory elements are correlated with the activity of SEs. The SNP rs2168101 has been demonstrated to affect neuroblastoma susceptibility; it is located in the *LMO1* superenhancer and changes a GATA-binding motif into a TATA motif, thus inhibiting the activity of the SE and decreasing the expression of *LMO1*^78^ (Fig. 3). Similarly, in diffuse large B-cell lymphoma, two SNPs (rs9831894 and rs6773363) of susceptibility loci have been identified and are a part of a tumor-specific SE^79^.

It has been shown that structural variations can disrupt 3D genome organization and active SE regions, known as “superenhancer hijacking”, which has been reported in neuroblastoma, leukemia, and colorectal cancer^80–85^. Superenhancer hijacking has been described as a novel mechanism for tumorigenesis in which chromosomal rearrangements cause enhancers to transfer to nearby oncogenes, thereby leading to high expression of oncogenes and the tumor initiation^82^. In colorectal cancer, *IGF2* is a target of enhancer hijacking, which is mediated by the formation of a contact domain comprising *IGF2* and a lineage-specific superenhancer^82^ (Fig. 3). In addition, Wang et al. developed a computational framework called NeoLoopFinder to predict enhancer hijacking by identifying the chromatin interactions induced by structural variations^80^. They found that enhancer hijacking-driven oncogenes such as *MYC*, *ETV1*, *PVT1*, and *CDK12* are significantly upregulated in cancer cells.

Viral infections have also been shown to induce SE formation and drive aberrant transcription of genes involved in the initiation and progression of tumors. Epstein‒Barr virus (EBV), human papilloma virus (HPV), human T-cell leukemia virus (HTLV), and human hepatitis B virus (HBV) are common oncogenic viruses, and their capacity to activate SE-associated genes has been reported in several tumors. HTLV-1 is associated with adult T-cell leukemia/lymphoma (ATLL) and specifically encodes the TF HBZ. HBZ binds to ATLL-specific *BATF3* superenhancers, leading to the expression of *BATF3* and other downstream genes and promoting the proliferation of ATLL cells^86^ (Fig. 3). In HPV-infected cervical cancer, integration of the viral genome into the host genome generates a superenhancer that drives the overexpression of viral E6 and E7, promoting the growth of cancer cells^87^.

## Clinical applications related to SEs in cancer

### Small-molecule inhibitors targeting SEs for tumor therapy

The high transcriptional activity of superenhancers mediates transcriptional addiction in aggressive tumors, making oncogenes extremely susceptible to transcriptional alterations^88^. Thus, targeting SE complexes with small-molecule inhibitors is a promising strategy for tumor therapy (Fig. 1). Unlike genetic mutations, epigenetic alterations are reversible, and many drugs targeting epigenetic regulators have exhibited therapeutic potential in clinical trials^89^. The sensitivity of SEs to emerging small-molecule inhibitors has been confirmed in various tumors, suggesting that SEs might be antitumor targets^90^. Some drugs have entered clinical trials (Table 1), with the hope that they can be applied clinically.

**Table 1:** Small-molecule inhibitors targeting SEs in clinical trials.

Target	Inhibitors	Tumor types	Clinical trial
BET proteins	BMS-986158	Advanced solid tumors or hematologic malignancies	NCT02419417 (Phase I/IIa)
	BMS-986158, BMS-986378 (CC-90010)	Pediatric cancers	NCT03936465 (Phase I)
	RO6870810	Advanced multiple myeloma	NCT03068351 (Phase Ib)
	CPI-0610	Multiple myeloma	NCT02157636 (Phase I)
	CPI-0610	Progressive lymphoma	NCT01949883 (Phase I)
	CPI-0610	Malignant peripheral nerve sheath tumors	NCT02986919 (Phase II)
	MK-8628 (OTX015)	Advanced solid tumors	NCT02259114 (Phase I)
	PLX51107	Acute myeloid leukemia	NCT04022785 (Phase I)
	SYHA1801	Advanced solid tumors	NCT04309968 (phase I)
	ZEN-3694	NUT carcinoma	NCT05019716 (Phase I/II)
	AZD5153	Malignant peripheral nerve sheath tumors	NCT05253131 (Phase I/II)
	SF1126	Advanced hepatocellular carcinoma	NCT03059147 (Phase I)
	ZEN-3694	Metastatic castration-resistant prostate cancer	NCT04471974 (Phase II)
	ZEN-3694	Advanced and refractory solid tumors and lymphomas	NCT05053971 (Phase Ib/II)
	ZEN003694	Triple-negative breast cancer	NCT05111561 (Phase I)
	FT-1101	Acute myeloid leukemia, non-Hodgkin lymphoma	NCT02543879 (Phase I)
	TQB3617	Advanced malignant tumors	NCT05110807 (Phase I)
	ZEN003694	Recurrent ovarian cancer	NCT05071937 (Phase II)
	ZEN003694	NUT carcinoma	NCT05019716 (Phase I/II)
	ZEN003694	Metastatic castration-resistant prostate cancer	NCT02711956 (Phase Ib/IIa)
	RO6870810 (TEN-010)	Acute myeloid leukemia	NCT02308761 (Phase I)
	CC-90010	Advanced solid tumors and relapsed/refractory non-Hodgkin lymphomas	NCT03220347 (Phase I)
CDK7	SY-5609	Advanced solid tumors	NCT04247126 (Phase I)
	XL102	Advanced or metastatic solid tumors	NCT04726332 (Phase I)
	CT7001	Advanced malignancies	NCT03363893 (Phase I/II)
CDK9	AZD4573	Hematological malignancies	NCT03263637 (Phase I)
	PRT2527	Advanced solid tumors	NCT05159518 (Phase I)
	TP-1287	Advanced solid tumors	NCT03604783 (Phase I)
	GFH009	Hematological malignancies	NCT04588922 (Phase I)
	Fadraciclib (CYC065)	Advanced solid tumors and lymphoma	NCT04983810 (Phase I/II)
	BAY1251152	Hematological malignancies	NCT02745743 (Phase I)
	TG02	Glioblastoma	NCT03224104 (Phase Ib)
CDK8	TSN084	Advanced malignant tumors	NCT05300438 (Phase I)
	RVU120	Acute myeloid leukemia	NCT04021368 (Phase I)
	BCD-115	Breast cancer	NCT03065010 (Phase I)

BRD4, a member of the bromodomain and extraterminal domain protein (BET) family, plays a role in SE organization and oncogene expression regulation^91^. Mechanistically, BRD4 binds to acetylated lysines in enhancers, SEs, and TFs, bringing them together and mediating transcriptional activation and elongation via RNA pol II and mediators^92,93^. Inhibition of BRD4 disrupts the communication between SEs and their target promoters, resulting in subsequent repression of oncogenes^91^. KDM6A, a histone demethylase, is frequently mutated, which promotes tumorigenesis. KDM6A loss was found to regulate aberrant activation of SEs of oncogenes, ultimately leading to pancreatic cancer development. KDM6A-deficient pancreatic cancer is sensitive to BET inhibitors (such as JQ1 and I-BET151), which can decrease the expression of SE-associated genes and suppress tumor growth^94^. Another BET inhibitor, OTX015, is effective against mouse and human MYCN-driven neuroblastoma in models, as it can selectively disrupt the binding of BRD4 and SEs and lead to the repression of MYCN expression^95^.

In addition, cyclin-dependent protein kinases (CDKs) are protein-serine/threonine kinases that play an essential role in regulating the cell cycle and transcription^96^. During transcriptional activation, BRD4 binds to SEs, followed by the recruitment of the TFIIH/CDK7 initiation complex and P-TEFb elongation complex containing CDK9^75^. Cyclin-dependent kinase 7 (CDK7), an important CDK, can phosphorylate the RNA pol II C-terminal domain (CTD) at serine 5 (Ser5) and Ser7, leading to transcriptional initiation^97^. CDK7 also phosphorylates and activates CDK9/cyclin T, and CDK9 increases the phosphorylation of RNA pol II at Ser2, thus promoting transcriptional elongation^98^. Therefore, given that CDK7 and CDK9 are directly or indirectly involved in cellular transcriptional regulation, inhibition of CDK7 and CDK9 may interfere with transcription^99^. Covalent CDK7 inhibitors (such as THZ1 and THZ2) result in the downregulation of oncogene transcription and can serve as SE blockers to inhibit the expression of SE-driven oncogenes^100^. THZ2 is a newly developed CDK7 inhibitor with a fivefold longer half-life than THZ1, and its antitumor effects have been demonstrated in triple-negative breast cancer, gastric cancer, and osteosarcoma^88,101,102^. Moreover, the CDK9 inhibitor AZD4573 has shown effective antitumor activity through inhibition of CDK9, and AZD4573 is currently being evaluated in phase I clinical trial for patients with hematological malignancies (NCT03263637)^103,104^.

More importantly, studies have shown that combination of SE inhibitors with traditional therapy enhances efficacy^105,106^. The notable antitumor effects of JQ1 and THZ1 have been demonstrated in multiple types of tumors, including triple-negative breast cancer, diffuse large B-cell lymphoma, and pancreatic cancer^88,107,108^. Simultaneous inhibition of BRD4 and CDK9 with JQ1 and LDC067 suppresses cell growth and migration in medulloblastoma^109,110^. The coinhibition of these two molecules also shows a similar antiproliferative effect in malignant rhabdoid tumors^109,110^. Combination therapy with JQ1 and the novel CDK7 inhibitor YKL-5-124 shows a synergistic effect in neuroblastoma and can delay resistance to BRD4 inhibition^111^. In addition, combination of a histone deacetylase (HDAC) inhibitor and JQ1 or OTX015 results in stronger repression of oncogenes and higher expression of tumor suppressor genes, suggesting that these epigenetic drugs have synergistic antitumor effects^112^. Similarly, when THZ1 is combined with an HDAC inhibitor, sensitivity notably increases^105,113^. Given the potential therapeutic effects of small-molecule inhibitors, rational combinational strategies may be more effective for cancer treatment.

Despite significant inhibitory effects in previous studies, some side effects of BRD4 or CDK7 inhibitors have been reported in animal experiments and phase I clinical trials, such as heart toxicity, gastrointestinal toxicity, fatigue, thrombocytopenia, and impairment of muscle function^114–116^. Furthermore, drug resistance is a challenge related to small-molecule inhibitors. In *MYCN*-driven neuroblastoma cells, upregulation of the multidrug transporters ABCB1 and ABCG2 results in resistance to THZ1^117^. In tumors with resistance to BRD4 inhibitors, hyperphosphorylation of BRD4 and epigenetic plasticity contribute to decreased sensitivity of JQ1^118,119^. The safety and efficacy of these small-molecule inhibitors, including potential side effects, off-target effects, and drug resistance, need to be further studied before clinical application.

### Utility of SEs in tumor prognostication

SEs can also serve as biomarkers for evaluating the prognosis of tumor patients. In HCC, the SE-associated lncRNA HCCL5 regulates malignant biological behavior and is associated with the prognosis of patients with HCC^50^. Further analysis revealed that the aberrant SE landscape in HCC is the result of extensive reprogramming. The key components of SEs (BRD4, CDK7, p300, and MED1) are overexpressed in HCC, which is associated with the poor prognosis of patients^120^. In addition, SEs with broad and high H3K27ac signals have been identified in nasopharyngeal carcinoma and were found to be associated with overexpression of the *ETV6* oncogene and poor prognosis^121^. Xu et al. defined an SE-associated gene risk signature to predict the response to chemotherapy in patients with diffuse large B-cell lymphoma, which may help clinicians make more appropriate treatment decisions^122^. Moreover, in our previous studies, we constructed prognostic models based on SE-associated genes to predict overall survival for osteosarcoma and multiple myeloma patients, which may be helpful for clinical treatment^123,124^. Therefore, oncogenic SEs may promote the malignancy of tumors and have utility in predicting the clinical outcome of tumor patients.

## The oncogenic roles and regulatory mechanisms of SEs in brain tumors

### SEs in glioblastoma

Glioblastoma (GBM), a high-grade glioma (World Health Organization grade IV), is the most common malignant brain tumor and has a 5-year survival rate of 5.6%^125^. Despite multimodal treatment, including surgical resection, radiation, and standard therapy with temozolomide (TMZ), the prognosis is universally poor due to therapy resistance and recurrence^126–128^. Therefore, it is urgent to explore the molecular mechanisms underlying GBM progression and develop novel therapeutic strategies to improve the prognosis of GBM patients.

#### Identification of SEs in GBM

In the past few years, several novel epigenetic markers contributing to the pathogenesis of GBM have been reported. In 2014, a population-based single-cell whole-genome sequencing methodology was applied to characterize genomic heterogeneity in EGFR-amplified GBM^129^. Researchers identified a translocation of a superenhancer to the 5’ promoter region of *TERT*, which can activate *TERT*^129^. Moreover, another study described 3D genome information through Hi-C sequencing in glioblastoma stem cells (GSCs)^130^. In GSCs, genomic structural variants lead to SE-promoter interactions, such as physical interactions between the two SEs (SE1 and SE2) and *JAK1*^130^. In addition, integration of Hi-C and chromatin data revealed strong H3K27ac signals in stemness genes and identified a region overlapping *SOX2* as an SE locus^130^. Currently, the majority of biopsies are preserved as archived formalin-fixed paraffin-embedded (FFPE) samples. Zhao et al. assessed FFPE tissue with antibody-guided chromatin tagmentation with sequencing (FACT-seq), the first highly sensitive method to describe histone modifications in FFPE tissues^131^. Using FACT-seq of H3K27ac in FFPE human GBM samples, the researchers identified 492 disease-specific SEs (in genes such as *EGFR*, *ETV1*, and *CDK6*)^131^. In summary, the development of novel technologies allows the identification of complete SE landscapes and histone modifications in the whole genome, which aids the investigation of the epigenetic regulation and heterogeneity of GBM.

#### SE-mediated aberrant transcriptional programs

**Fig. 4:**
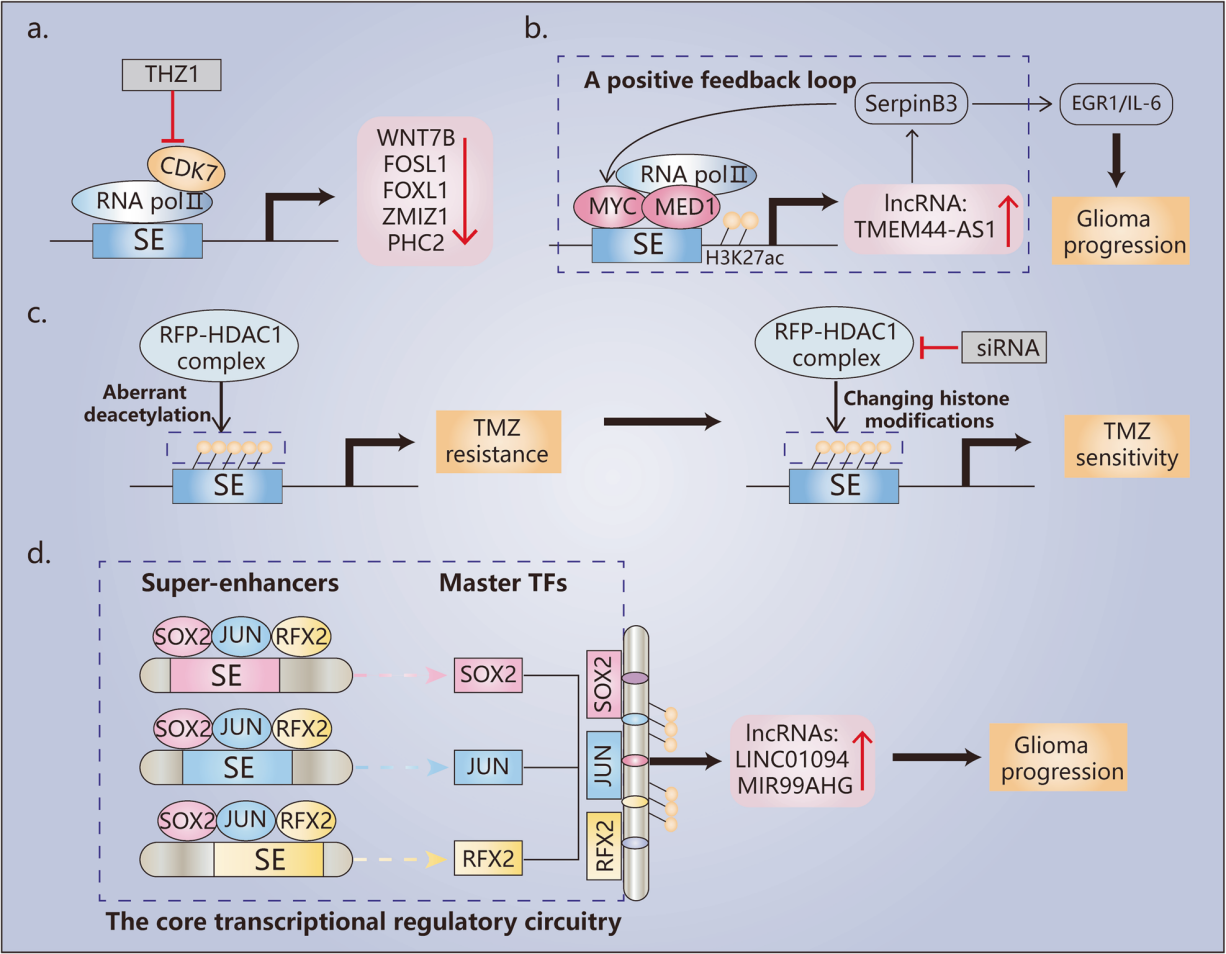
Regulatory mechanisms of oncogenic SEs in glioblastoma (GBM). **a** In GBM cells, five highly expressed SE-associated genes are associated with sensitivity to the CDK7 inhibitor THZ1. **b** Myc and MED1 mediate the epigenetic activation of TMEM44-AS1, which is directly bound to SerpinB3, and sequentially activate Myc and EGR1/IL-6 signaling; Myc induces transcription of TMEM44-AS1 and binds to the SE region, forming a positive feedback loop with TMEM44-AS1, thus aggravating tumor progression **c** The RFP-HDAC1 complex contributes to TMZ resistance via aberrant deacetylation of H3K27ac, and the disruption of the complex leads to an increase in TMZ efficacy by changing core histone modifications in GBM. **d** The core transcriptional regulatory circuitry (CRC) in GBM: SE-driven TFs are highly enriched in SE regions and regulate the expression of SE-associated lncRNAs. The BET degrader dBET6 can effectively disrupt the expression of core TFs.

Research focused on SE-driven aberrant transcriptional programs in GBM is gradually increasing. Since CDK7 can phosphorylate RNA Pol II to initiate transcription, CDK7 expression is significantly increased in GBM and is associated with poor prognosis^98,132^. The CDK7 inhibitor THZ1 can disrupt global gene transcription and preferentially target SE-associated genes in GBM cells. For example, five highly expressed SE-associated genes (*WNT7B*, *FOSL1*, *FOXL1*, *ZMIZ1*, and *PHC2*) were found to be associated with sensitivity to THZ1, and their knockdown inhibited the proliferation of GBM cells (Fig. 4a)^132^. In addition, GBM cells harbor a superenhancer in the Mcl-1 locus, leading to an increased level of Mcl-1, a member of the antiapoptotic Bcl-2 family of proteins^133^. Pharmacological inhibition of the SE by THZ1 decreased Mcl-1 mRNA and protein levels. A similar study identified the SE-derived lncRNA TMEM44-AS1, which was upregulated and correlated with malignant phenotypes in GBM^134^. When Myc directly binds to the promoter and SE of TMEM44-AS1 and colocalizes with MED1, H3K27ac, and RNA pol II, TMEM44-AS1 is activated^134^. The Myc inhibitor Myci975 can inhibit the growth of GBM cells by inhibiting the Myc/TMEM44-AS1 feedback loop (Fig. 4b)^134^. Together, these findings suggest that SE-mediated aberrant transcriptional programs are associated with GBM progression, and targeting these oncogenic SEs may be an effective therapeutic strategy for GBM.

#### SE-driven mechanisms of chemoresistance

Resistance to TMZ occurs in almost all patients during chemotherapy, leading to a poor prognosis. High expression of O6-methylguanine-DNA methyltransferase (MGMT) is one of the leading causes of TMZ resistance because MGMT is a DNA repair factor that can reverse DNA damage caused by TMZ^135^. MGMT expression is negatively associated with methylation modification in the *MGMT* promoter region^136^. Thus, the higher the methylation degree of the *MGMT* gene promoter region in GBM patients is, the better the effect of TMZ. Apart from methylation, MGMT expression can be regulated by other factors. It was found that the enhancer (K-M enhancer) located between the *Ki67* and *MGMT* genes can increase MGMT expression and is activated in TMZ-resistant GBM tissues^137^. Deleting the K-M enhancer reduces MGMT and Ki67 expression, thus increasing sensitivity to TMZ^137^. Moreover, another study demonstrated that aberrant transcriptional regulation of SEs is a cause of resistance to TMZ^138^. The H3K27ac status regulates *cis*-regulatory elements such as SEs. However, the RET finger protein-histone deacetylase 1 (RFP-HDAC1) complex deacetylates H3K27ac marks on core histones, decreasing gene transcription levels^138^. Disruption of the HDAC complex can increase TMZ sensitivity in GBM cells by affecting the H3K27ac status (Fig. 4c)^138,139^. In the future, more in-depth studies are needed to investigate whether the epigenetic regulatory mechanism underlying TMZ resistance is mediated by histone acetylation and which SEs drive transcriptional reprogramming. GBM resistance to TMZ may be related to abnormal SE activity, providing new ideas for overcoming GBM drug resistance.

#### HDACs regulate SE activity

Multiple lines of evidence have reported that the malignant phenotype of GBM is regulated by SEs and HDACs. HDAC1, together with BRD4, RNA pol II, and other key components, is enriched in SEs^10^. SLC30A3 is significantly reduced in GBM tissues and can inhibit the growth and metastasis of GBM cells^140^. Overexpression of HDAC1 decreases the H3K27ac level in the SE region of SLC30A3, which inhibits the expression of SLC30A3^140^. HDAC1 is predominantly associated with transcriptional repression, while the transcriptional regulation mechanism of HDAC7 is complex and related to both activation and repression. In stem-like breast cancer cells, HDAC1 and HDAC3 regulate the expression of HDAC7^141^. In turn, HDAC7 maintains H3K27ac levels by binding to SEs, thereby promoting the expression of SE-associated oncogenes^141^. HDAC1/3 knockdown increases the overall H3K27ac level, but HDAC7 knockdown decreases the H3K27ac level, and these changes are only observed in cancer stem cells and not in other cells^141^. These results suggest functional differences between HDAC1/3 and HDAC7. Inhibition of HDAC1/2/3 disrupts SE activity by increasing the accessibility of TF sites and disrupting chromatin loops, an indirect effect^105,142–144^. In GBM models, the HDAC inhibitors panobinostat and romidepsin have been shown to target SEs and elicit metabolic reprogramming through H3K27ac modification, thereby suppressing GBM progression^143,145^. Overall, different types of HDACs have different regulatory effects on SE activity.

The molecular mechanisms underlying HDAC inhibitor-mediated SE activity are not yet fully understood. Some studies have investigated the regulatory mechanisms of HDAC inhibitors: (1) reshaping epigenetic markers by promoting H3K27 acetylation^144,146,147^; (2) decreasing the binding of RNA pol II at SE regions^143–145^; (3) inducing SE looping defects by reducing contact with SE components and promoters^142^; and (4) reducing the expression of core regulatory circuitry TFs to repress SE activity^72^. These epigenetic alterations cause the global disruption of SEs and thus inhibit SE-associated transcript and gene expression^143,144^. Based on their antitumor effects, HDAC inhibitors should be able to exert synergistic effects in combination with SE inhibitors (such as THZ1 and JQ1) and chemotherapies in neuroblastoma, glioblastoma, and other tumors^72,105,112,113,146^.

#### SE-mediated core regulatory circuitry

SE-mediated core regulatory circuitry plays a crucial role in human cancers, including medulloblastoma, neuroblastoma, and glioblastoma^8,148–150^. An active chromatin landscape was mapped in GSCs, identifying SE-associated genes and core TFs that establish SEs and maintain GSC identity^151^. The study found that many genes defining GSC identity are highly expressed and regulated by SEs, such as *SOX2*, *SEPT9*, *CXXC5*, *CDK6*, *SALL3*, and *EGFR*, which are required for tumor cell proliferation^151^. In addition, another study depicted the core TFs and their interconnected regulatory circuitry in GBMs, showing that many motifs of SE-driven TFs (SOX2, JUN, and RFX2) are enriched in SE regions (Fig. 4d)^152^. The stem cell TF SOX2 can bind to the ELOVL2 superenhancer in GSCs to drive the expression of *ELOVL2*, which is critical for the maintenance of cancer stem cells^153^. Furthermore, nine SE-associated lncRNAs were identified in GBM tissues, and *LINC01094* is one of the lncRNAs whose disease-specific expression is regulated by tumor-associated core TFs and SEs^152^. SEs and the core regulatory circuitry in GBM cooperatively regulate transcriptional programs, which is significant for GBM progression.

#### Roles of seRNAs in gliomas

Some seRNA-regulated immune-related genes (IRGs) relevant in the tumor microenvironment were identified in glioma^154^, and they were used to construct a prognostic model that can predict the survival outcomes of patients with glioma. These glioma-specific seRNAs that regulate IRGs are located in adjacent sites and coexpressed in tumors, suggesting that they have potential as therapeutic targets^154,155^.

#### Promising antitumor effects of targeting BET proteins

The BET inhibitor I-BET151 inhibits the proliferation and eliminates the tumorigenicity of GBM cells^156–158^. The antiproliferative activity is equivalent to that of TMZ, suggesting that I-BET151 may be beneficial for GBM patients with TMZ resistance^156^. In addition, another BET inhibitor, OTX015, was found to cross the BBB and selectively penetrate tumor tissues in a preclinical study, indicating its potential efficacy in GBM^159^. In addition, dBET6, a chemical degrader of BET proteins, can inhibit the proliferation and self-renewal ability of GBM cells by significantly reducing BET protein occupancy, RNA pol II activity, and active histone markers, and dBET6 is superior to first-generation BRD4 inhibitors such as JQ1^160,161^. Interestingly, GBM cells exhibit sex-specific responses to the BRD4 inhibitor JQ1, which is correlated with sex-specific gene expression patterns. The sensitivity of male patient-derived GBM cells to BET inhibitors is higher than that of female patient-derived GBM cells^162^.

As newly recognized epigenetic elements, numerous SEs have been identified in GBM and have been shown to be related to tumor progression (Table 2). As described above, novel epigenetic mechanisms that regulate the malignant biological behaviors of GBM cells have been revealed, providing potential treatments. In the future, targeting SEs may be a promising complement to traditional therapies for GBM treatment.

**Table 2:** Summary of oncogenic SEs in brain tumors.

Type of brain tumor	Superenhancer-associated genes	Features of SEs or mechanisms of SE inhibitors	Malignant phenotypes	Inhibitors	References
GBM	SLC30A3	Significant modification and negative regulation of H3K27ac levels by SE-dependent HDAC1	Growth, metastasis, and apoptosis	/	^140^
GBM	TMEM44-AS1	A positive feedback loop between TMEM44-AS1 and Myc/MED1	Proliferation, colony formation, migration, and invasion	Myci975	^134^
GBM	MYC, HK2, and ENO1	HDAC inhibitors disrupt SEs of Warburg effect-related genes	Reprogramming of tumor cell oxidative metabolism	HDAC inhibitors (panobinostat and romidepsin)	^143,145^
GBM	TERT	Translocation of an SE within chromosome 10 to the TERT promoter		/	^129^
GBM	WNT7B, FOSL1, FOXL1, ZMIZ1, and PHC2	THZ1 inhibits global gene transcription and preferentially targets SE-associated genes	Proliferation	THZ1	^132^
GBM	SOX2, SEPT9, CXXC5, CDK6, SALL3, and EGFR	GSC identity genes are regulated by SEs and are required for GSC maintenance	Proliferation and growth	/	^151^
GBM	Mcl-1	Pharmacological inhibition of SEs decreases Mcl-1 mRNA and protein levels	Cell death induction with features of apoptosis	THZ1 and ABT263	^133^
MB	GFI1 and GFI1B	Genome rearrangement and enhancer hijacking		/	^167^
MB	ALK, MYC, SMO, and ETV4	MB superenhancers characterize subgroup-specific identity		/	^148^
MB	ARL4D	An OTX2-SE-ARL4D regulatory axis		JQ1 and THZ1	^168^
MB	DNMT3A, SIRT1, and BCL6	MLL4 loss promotes MB by decreasing SEs and H3K4me3 signals to downregulate tumor suppressor genes	Proliferation and tumorigenesis	/	^171^
DIPG	SOX10, OLIG1, MYRF, MYT1, and MBP	DIPG is vulnerable to transcriptional disruption via BRD4 or CDK7 blockade	Proliferation, invasion, and migration	Panobinostat (an HDAC inhibitor), JQ1, and THZ1	^105^
DIPG	c-MYC	Reprogramming of the enhancer landscape is induced by H3K27 hyperacetylation	Proliferation	ICG-001 (a CBP inhibitor) and JQ1	^174^
AT/RT	SMARCB1	SMARCB1 represses bivalent genes and antagonizes chromatin accessibility at SEs	Cell growth	/	^191,193^
ETMR	MYCN, LIN28A, and DNMT3B6	A C19MC-LIN28A-MYCN SE-dependent oncogenic circuit	Proliferation	JQ1	^196^
Meningioma	HOXD	HOXD is regulated by the TF FOXM1 and its superenhancer	Aggressiveness	/	^198^
Ependymoma	EPHB2 and CCND1	JQ1 inhibits the proliferation of ependymoma cells at nanomolar concentrations	Cell growth and proliferation	JQ1	^202^

*GBM* glioblastoma, *GSC* glioblastoma stem cell, *MB* medulloblastoma, *DIPG* diffuse intrinsic pontine glioma, *HDAC* histone deacetylase, *CBP* CREB-binding protein, *AT/RT* atypical rhabdoid/teratoid tumors, *ETMR* embryonal tumor with multilayered rosette.

### SEs in pediatric brain tumors

#### SEs in medulloblastoma

Medulloblastoma (MB) is the most common malignant brain tumor in children and adolescents younger than 15 years old^163^. Previous genomic studies have revealed four molecular subgroups of MB (WNT, sonic-hedgehog (SHH), Group 3, and Group 4) with different biological and clinical behaviors^164,165^. Among these subgroups, Group 3 and Group 4 MBs account for the majority of cases and have the poorest outcomes^166^.

A study of MB illustrated that oncogenic drivers were restricted to Group 3 and Group 4^167^. The growth factor independent 1 family oncogenes *GFI1* and *GFI1B* are oncogenes of Group 3 and Group 4 MBs that can cooperate with *MYC* to promote MB formation in vivo^167^. Genomic structural variants occur in the *GFI1* or *GFI1B* coding sequences proximal to enhancers and superenhancers, leading to oncogenic activation of GFI1 and GFI1B^167^. In addition, scientists have identified some subgroup-specific SEs in MBs that can activate oncogenes, including *ALK*, *MYC*, *SMO*, and *ETV4*^148^. In Group 3 MB, an SE-driven transcriptional regulatory network consisting of 14 SE-associated genes exists^168^. A BET inhibitor (JQ1) and a CDK7 inhibitor (THZ1) showed synergistic inhibitory effects, with therapeutic potential for Group 3 MB^168^. Moreover, some adult SHH MB patients present with truncating mutations in the chromatin reader BRPF1, which are absent or rare in pediatric patients^169^. Mutated BRPF1 increases the accessibility of a subset of SEs associated with key genes involved in cerebellum development and chromatin remodeling, promoting tumorigenesis in adult SHH MB^170^.

Superenhancers not only activate oncogene expression but are also associated with tumor suppressor gene activation. Brain-specific knockout of the H3K4 methyltransferase MLL4 in mice can induce MB^171^. Mechanistically, MLL4 loss leads to MB by reducing broad H3K4me3 and SE signals, downregulating tumor suppressors (Dnmt3a and Bcl6), and extensively damaging epigenomic signatures^171^. Therefore, MLL4-established superenhancers play an important role in tumor suppression in normal cells. The antitumor role of SEs has also been reported in other types of tumors, such as chronic myelogenous leukemia and breast cancer^172^. Superenhancers can drive either oncogene expression or tumor suppressor expression, playing dual roles in tumor progression. SE-associated genes and possible mechanisms in MB are shown in Table 2.

#### SEs in diffuse intrinsic pontine glioma

Diffuse intrinsic pontine glioma (DIPG) is a fatal pediatric brain tumor with a median survival time of 11 months^173^. Because of its diffuse growth and location, DIPG is inoperable, and the standard therapy is radiation therapy, but its effectiveness is extremely limited^174^. The characterization and development of drugs are challenging due to the scarcity of DIPG samples.

Approximately 85% of DIPGs are characterized by mutation of lysine 27 to methionine in histone 3 (H3K27M), which leads to oncogenic transcription dysregulation and increased stem-like potential and proliferation^105,173–175^. H3K27M glioma cells exhibit greater proliferation potential due to the aberrant oncogenic program^176^. Together with RNA pol II and H3K27ac, H3K27M localizes to transcriptionally active regions and can drive SE formation^177,178^. H3K27M inhibits the enzymatic activity of PCR2 through its interaction with the E2H2 subunit, resulting in the loss of H3K27me3 at SE regions^179,180^. H3K27M leads to a more accessible chromatin configuration in key regulatory regions, which can expose binding site motifs for key TFs, including ASCL1 and NEUROD1^181^. These TFs then bind to SEs to activate neurogenesis and NOTCH pathway-related genes, ultimately contributing to glioma formation^180,181^. Recent studies of H3K27M DIPG in vitro and in vivo have identified some important druggable targets and found some effective small molecules to reverse epigenetic alterations, including the HDAC inhibitor panobinostat^182^, the H3K27me2/3 inhibitor GSKJ4^182^, the BET inhibitor JQ1^178^, and the selective E2H2 inhibitor EPZ6438^183^.

The analysis of SEs in DIPG has revealed potential cell-identity genes, supporting the idea that DIPG originates from a precursor cell of the oligodendroglial lineage. These SE-associated genes include genes classically associated with oligodendrocyte precursor cells (such as *SOX10*) and genes expressed by oligodendroglial lineage cells during differentiation (such as *OLIG1*, *MYRF*, *MYT1*, and *MBP*)^105^. DIPG is vulnerable to BRD4 and CDK7 blockade, which can impair DIPG cell growth^105^. For example, the BRD4 inhibitor JQ1 can induce neuron-like differentiation and delay tumor growth in a mouse model of DIPG, and the CDK7 inhibitor THZ1 can disrupt transcription and inhibit DIPG growth^105,184^. Hyperacetylation in DIPG favors the action of BDR4 and leads to enhancer landscape reprogramming, activating SE-driven oncogenes in DIPG^174^. In addition, CBP is related to transcription activation, and its activity can be enhanced by BRD4^185^. The combination of JQ1 and a CBP inhibitor (ICG-001) can reverse the detrimental SE programs activated by BET or CBP^174^. Collectively, these findings suggest that SE-driven oncogenes in DIPG can be targeted with SE blockers. SE-associated genes and possible mechanisms in DIPG are shown in Table 2.

#### SEs in other rare pediatric brain tumors

DNA 5-hydroxymethylcytosine (5hmC), one of the molecular alterations in GBM, can recruit DNA-binding proteins and is essential for GBM tumorigenesis^186^. 5hmC preferentially localizes to the enhancers and superenhancers of tumor-specific genes in glioblastoma, activating disease-specific gene expression programs^187^. 5hmC alteration is observed not only in adult brain tumors but also in pediatric brain tumors according to a recent study; 5hmC is located in TF binding sites and SE regions and is crucial to cell identity^187,188^. Thus, the epigenetic alterations of SEs in adult and pediatric brain tumors are somewhat similar, and 5hmC can be used to identify these aberrant regulatory elements.

Atypical rhabdoid/teratoid tumors (AT/RTs) are aggressive and lethal cancers that can be diagnosed at a young age^189^. The SWItch/Sucrose Non-Fermentable (SWI/SNF) chromatin-remodeling complex is an essential regulator of pluripotency in human embryonic stem cells. Nevertheless, mutation of its core subunit SMARCB1 can lead to AT/RTs due to the disruption of enhancer accessibility^190^. With noticeable H3K27ac features in enhancer regions, SMARCB1 is required for the integrity of the SWI/SNF complex^191^, which is inactivated abnormally in most rhabdoid tumors^192^. Loss of SMARCB1 reduces genome-wide targeting at enhancers, thus impairing the functions of the SWI/SNF complex. However, the small amount of residual SWI/SNF complexes preferentially bind to SEs, including some shared by all subtypes, such as SPRY1, and other lineage-specific SEs, such as SOX2, in brain-derived rhabdoid tumors, which is crucial for maintaining aberrant cell identity. Then, these SEs drive oncogenic transformation by locking cells into a poorly differentiated and highly proliferative state^191^. Further research found that knockdown of SMARCB1 prevents silencing in SE regions, thus leading to transcriptional upregulation in human embryonic stem cells^193^. Therefore, SMARCB1 and its regulatory effects on SEs in AT/RT are part of a novel AT/RT tumorigenesis mechanism.

Embryonal tumor with multilayered rosettes (ETMR) is a sporadic and difficult-to-treat brain tumor in infants and young children, with rapid progression and only 10–20% overall survival^194,195^. Because there are few research models, mechanistic and therapeutic studies of this rare disease are extremely limited. Recently, an oncogenic circuit driven by hijacked superenhancers in ETMRs was revealed. The C19MC-TTYH1 gene fusion and MYCN DNA interactions create superenhancers. Then, the interaction between C19MC-TTYH1 superenhancers and MYCN enhancers fortifies the C19MC-LIN28A-MYCN circuitry, driving the expression of embryonically restricted DNMT3B6 to promote a primitive malignant epigenetic state in ETMRs^196^. Interestingly, the BET inhibitor JQ1 can downregulate key components of the C19MC-LIN28A-MYCN circuit, including MYCN, LIN28A, and DNMT3B6, disrupting the circuit and inducing ETMR cell death^196^. The unique SE-dependent oncogenic circuit protects the ETMR and is vulnerable to BET inhibition. Therefore, inhibition of BET may be a promising therapeutic strategy for this orphan disease. SE-associated genes and possible mechanisms in these pediatric brain tumors are shown in Table 2.

### SEs in other brain tumors

Meningioma is one of the most common intracranial tumors. Most meningioma patients can be cured by surgical resection, but approximately 20% of patients experience an aggressive clinical course with tumor recurrence or progression^197^. A comprehensive investigation of the genomic landscape has revealed the overall genomic instability in aggressive meningioma^198^. Upregulation of the SE-associated *HOXD* gene is associated with meningioma aggressiveness^198^ (Table 2).

Ependymoma is a rare disease that can arise throughout neuraxis^199^. In children, ependymomas mainly occur intracranially, while in adults, the spine is the most common location of ependymomas^199^. The challenges of ependymomas are resistance to chemotherapy and lack of effective molecular targets. More than 60% of ependymomas harbor a *ZFTA-RELA* (ZFTA^fus^) gene fusion^200^. ZFTA^fus^ contributes to an oncogenic transcriptional program because it binds TF motifs and recruits transcriptional coactivators (BRD4, EP300, CBP, RNA pol II), thus driving SE gene expression in ependymoma^201^. To identify SE-associated genes that ependymoma cells depend on, Mack et al. analyzed the chromatin landscapes of ependymomas^202^. In two cohorts of ependymoma specimens, they identified that the vast majority of SEs were tumor-specific and enriched in oncogenes. Among the genes, *EPHB2* and *CCND1* have been previously proven to be ependymoma-related oncogenes^203,204^. In addition, ependymoma is sensitive to the BET inhibitor JQ1, which can inhibit the proliferation of ependymoma cells^202^ (Table 2).

## Concluding remarks

The discovery of SEs is a novel breakthrough in the field of epigenetics. SEs are core regulatory elements that maintain the identity of cancer cells and drive cancer cells to become highly addicted to oncogene transcription. SEs regulate the expression of oncogenes that facilitate proliferation, migration, invasion, and even drug resistance, thus promoting tumor malignancy. With the accumulation of relevant research, the role of SEs in brain tumors is becoming increasingly clear. However, the composition of tumor-specific SEs and their potential molecular mechanisms remain to be further investigated.

Transcriptional activators (such as BRD4 and CDK7) are highly enriched in oncogenic SE regions, and their inhibition preferentially affects SE-associated genes in tumor cells. Some inhibitors targeting transcriptional activators have been evaluated in clinical trials. Moreover, combining SE inhibitors with other chemotherapeutic drugs can suppress tumor growth, providing a new strategy for cancer treatment. Since transcription is a basic biological process common to all nucleated cells, targeting SE-associated transcription may lead to general toxicity. Although inhibitors targeting SEs, including JQ1, THZ1, and THZ2, have been studied in many tumors, their potential side effects and off-target effects need further study.

As described above, the development of various brain tumors is closely associated with SEs and downstream oncogenes, and SEs may serve as master gene regulators and novel therapeutic targets. Further studies on the underlying mechanisms of SE activation will shed light on the complex pathogenesis of brain tumors. With persistent SE research efforts, breakthroughs in the treatment of malignant brain tumors are expected.
